# Identification of avoidable patients at triage in a Paediatric Emergency Department: a decision support system using predictive analytics

**DOI:** 10.1186/s12873-024-01029-3

**Published:** 2024-08-18

**Authors:** João Viana, Júlio Souza, Ruben Rocha, Almeida Santos, Alberto Freitas

**Affiliations:** 1 CINTESIS – Centre for Health Technology and Services Research, University of Porto, Porto, Portugal; 2 Department of Community Medicine, Information and Health Decision Sciences, Faculty of Medicine of the University of Porto Al. Prof. Hernâni Monteiro, Porto, 4200 - 319 Portugal; 3 Institute of Engineering – Polytechnic of Porto, Porto, Portugal; 4 Serviço de Pediatria / Urgência Pediátrica, UAG da Mulher E da Criança, Centro Hospitalar Universitário de São João, Porto, Portugal; 5 Departamento de Ginecologia-Obstetrícia e Pediatria, Faculty of Medicine of the University of Porto, Porto, Portugal

**Keywords:** Paediatrics, Triage, Emergency Services, AI, Machine Learning, Fast Track, Management

## Abstract

**Background:**

Crowding has been a longstanding issue in emergency departments. To address this, a fast-track system for avoidable patients is being implemented in the Paediatric Emergency Department where our study is conducted. Our goal is to develop an optimized Decision Support System that helps in directing patients to this fast track. We evaluated various Machine Learning models, focusing on a balance between complexity, predictive performance, and interpretability.

**Methods:**

This is a retrospective study considering all visits to a university-affiliated metropolitan hospital’s PED between 2014 and 2019. Using information available at the time of triage, we trained several models to predict whether a visit is avoidable and should be directed to a fast-track area.

**Results:**

A total of 507,708 visits to the PED were used in the training and testing of the models. Regarding the outcome, 41.6% of the visits were considered avoidable. Except for the classification made by triage rules, i.e. considering levels 1,2, and 3 as non-avoidable and 4 and 5 as avoidable, all models had similar results in model’s evaluation metrics, e.g. Area Under the Curve ranging from 74% to 80%.

**Conclusions:**

Regarding predictive performance, the pruned decision tree had evaluation metrics results that were comparable to the other ML models. Furthermore, it offers a low complexity and easy to implement solution. When considering interpretability, a paramount requisite in healthcare since it relates to the trustworthiness and transparency of the system, the pruned decision tree excels.

Overall, this paper contributes to the growing body of research on the use of machine learning in healthcare. It highlights practical benefits for patients and healthcare systems of the use ML-based DSS in emergency medicine. Moreover, the obtained results can potentially help to design patients’ flow management strategies in PED settings, which has been sought as a solution for addressing the long-standing problem of overcrowding.

## Introduction

Emergency Department (ED) crowding occurs when demands are greater than the hospitals’ capacity to ensure timely care in the ED. This is a multifactorial problem with multiple solutions. These problems can be tackled by influencing demand e.g., implementing gatekeeping policies, or optimizing the service provided. Furthermore, ED overcrowding and high patient volumes can result in delays in care, suboptimal treatment decisions, and increased risk of adverse events, including mortality, both in paediatric and adult settings [1–3]. To address these challenges, various approaches have been proposed. One of the most studied is the alteration of patient flow i.e., the implementation of fast tracks, based on the severity of the patients’ condition. This approach have shown to be able to improve both efficiency and outcomes, as well as reducing waiting times and overcrowding [4, 5].

Accessing the patients’ severity i.e. the identification of clinically divertible attendances or clinically unnecessary attendances patients [6], to be steered to fast tracks, has been made mostly using triage levels [7–10] or empirical rules [11]. The validity and accuracy of the identification of these avoidable visits is paramount [12], since it evolves not only patient flow efficiency but also patient safety. On the other hand, the emergence of robust machine learning (ML) algorithms has shown potential to improve predictive ability of various outcomes [13–16], which in turn could be leveraged to aid in the identification of these avoidable visits [16, 17].

In the hospital this study is being conducted, the Paediatric Emergency Department (PED) is undergoing restructuring, which includes the introduction of a fast-track system. Consequently, there’s a need to develop an algorithm to identify patients who are suitable for this expedited care pathway. A system that identifies these patients accurately has the potential to reduce the amount of time patients spend in the ED, reducing departmental crowding and ultimately support better patient outcomes. It will also, in all likelihood, help the reduction of ED overcrowding and facilitate a more effective allocation of healthcare resources.

Hence, we aim to create a data-driven, optimized DSS to aid in the selection of patients deemed avoidable and redirect them for a fast track. To achieve this, we compared different ML models and approaches, considering the balance between implementation complexity, predictive performance, and interpretability.

## Methods

### Study design, setting and participants

This study was an observational and retrospective research, conducted in a university-affiliated metropolitan hospital's PED. The hospital serves a population of about 800,000 and receives an average of 76,000 visits annually from an estimated 137,016 children or adolescents aged 0 to 17 years [18].

In the PED, there are 4–5 physicians and 7–8 nurses, working in 12-h shifts to ensure 24/7 coverage.

In this study, all presentations made to the hospital’s PED (i.e., from 0 to 17 years old) in a 4-year period (between 01/Jan/2016, and 31/Dec/2019) were considered.

The PED nursing team triages visitors according to the Canadian Triage and Acuity Scale paediatric guidelines (PaedCTAS), which is structured around evaluating physiological factors, including appearance, neurological status, respiratory rate, heart rate, and perfusion, alongside presenting symptoms to determine triage levels. Similar to the adult version of the Canadian Triage and Acuity Scale (CTAS), the PaedCTAS delineates five levels of triage i.e. Level 1(Red)—“Resuscitation”, Level 2 (Orange)—“Emergent”, Level 3 (Yellow)—“Urgent”, Level 4 (Green)—“Less Urgent” and Level 5 (Blue)—“Non Urgent”. These levels reflect the severity and urgency of the patient's condition, target times for medical assessment and intervention, and provide examples of typical clinical presentations and critical diagnoses [19].

This paper follows the structure presented in the RECORD statement i.e. The REporting of studies Conducted using Observational Routinely-collected health Data [20].

### PED restructuring

**Fig. 1:**
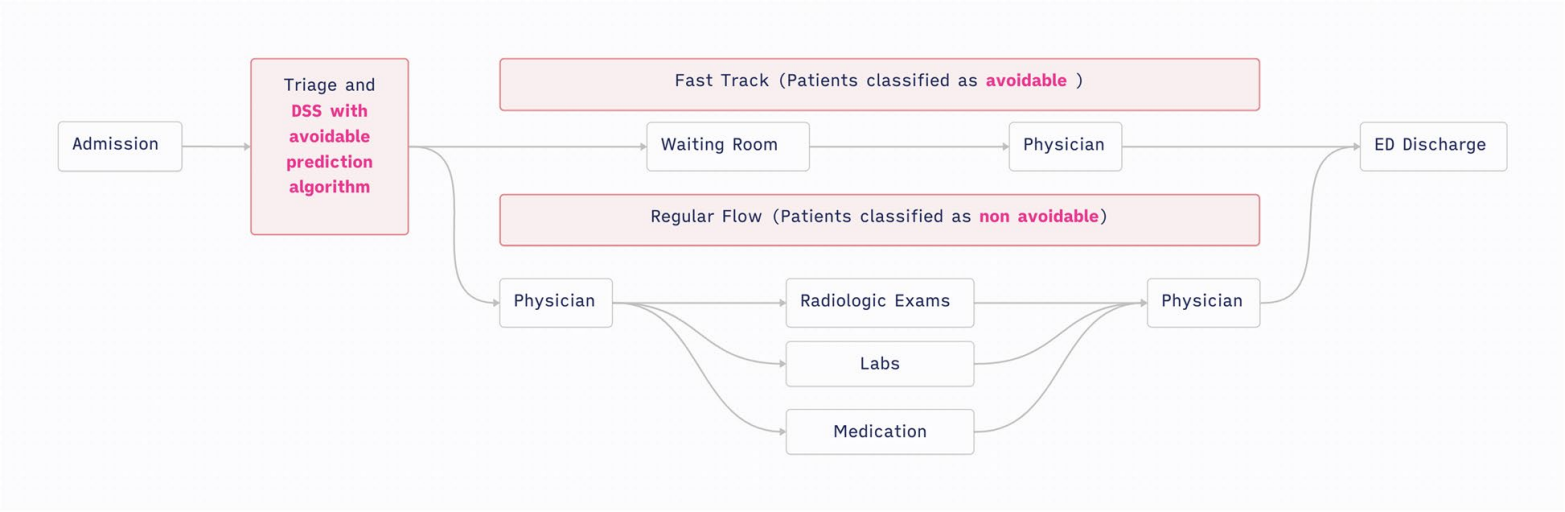
Schema of the restructuring of the Paediatric Emergency department regarding avoidable patient flow

The hospital's PED is undergoing a significant restructuring.. Along with this restructuring, a fast track for avoidable patients is to be implemented. A schema of the fast-track configuration is presented in Fig. 1.

### Data collection

All patient data is registered using a proprietary information system called JOne, where events are logged e.g. attending medical staff, diagnoses and cause of admission. All sociodemographic information and triage procedures are also registered in this information system.

All data access permissions, i.e., from the hospital board of directors, hospital epidemiology centre, information access officer and ethical committee were granted for this study. [FMUP 180/18].

### Outcome

The dependent variable was defined by the conjunction of several PED markers, resulting in a restrictive definition of avoidable. A PED visit is considered avoidable if the patient is discharged home and no diagnostic tests (i.e., blood tests and radiology exams), procedures or medications were required during the stay. The patients were also not asked to stay in the ED, for the physician to better assess the condition’s evolution. In summary, an avoidable visit is done by a patient that only has contact with the physician and is discharged home. The bullet list below summarizes the approach taken in the construction of the outcome to be predicted.

- Avoidable visit;
  - ◦ The patient was not medicated
  - ◦ The patient did not undergo any radiologic exams
  - ◦ The patient did not undergo any blood analysis
  - ◦ The patient did not stay for observations
  - ◦ The patient was discharged home

### Predictors and feature selection

A list of variables known at the time of the patient’s triage were used as predictors. Feature selection was done on a model-by-model basis, since associations between variables, redundant variables and low variance variables are handled differently depending on the model.

- Age
- Sex
- Season – Season of the year of patient’s arrival to the PED
- Month – Month of the year of patient’s arrival to the PED
- Day of week –weekday of the patient’s arrival to the PED
- Hour of day – Time of day of patient’s arrival to the PED, in. hourly slots
- Pretriage discriminator group –Paediatric Assessment Triangle group selection as described in PaedCTAS [19]
- Pretriage discriminator –Paediatric Assessment Triangle selection as described in PaedCTAS [19]
- Main complaint group—Triage complaint group as described in PaedCTAS [19]
- Main complaint discriminator – Triage complaint as described in PaedCTAS [19]
- Residence municipality – patient’s residence, municipalities outside the catchment area were residual and were grouped as other
- Triage level – PaedCTAS level of triage
- Referral – A referral patient was defined as ‘not walk-in’ patient e.g. referral from PCP, private clinic or other hospitals
- Made return visit X hours prior- Patient made at least one visit to the PED X (i.e.12, 24, 48, 72) hours prior to current visit i.e. the current visit is a return visit
- Visit by frequent attender – Visit made by frequent attender i.e. > 4 visits per year

### Machine learning models

Several ML models were created to evaluate the appropriateness for implementation in this context. These models ranged from simple rules, based on triage level (i.e. visits triaged levels 1, 2 and 3 are directed to regular emergency department patient flow while visits triaged levels 4 and 5 are directed to fast track), to a neural network. The appropriateness involved three dimensions: (1) complexity, some models could be implemented as simple rules, others need to be integrated into the hospital’s information system; (2) interpretability, can the reasoning behind the decision be understood i.e., glass box model or not i.e., black box, and (3) predictive performance. All the models used in this study and their classifications are enumerated in Table 1.

**Table 1:** List of classifications models used to predict if a visit to the Paediatrics emergency department is avoidable and should be directed the fast-track area

Model	Type	Interpretability	Implementation	R Package
Split made by triage levels^1^	Rules	Glass box	Paper based	Base R
Simple classification tree^2^	Tree based	Glass box	Paper based	rpart
Logistic regression	Regression	Glass box	Integrated in HIS	glm
Naive bayes	Bayesian	Glass box	Integrated in HIS	klaR
Complex classification tree	Tree based	Glass box	Integrated in HIS	rpart
Random forest	Tree based ensemble	Black box	Integrated in HIS	ranger
XGboost [21]	Tree based ensemble	Black box	Integrated in HIS	xgboost
Tabnet [22]	Deep learning	Black box	Integrated in HIS	torch
Tensor Flow through Keras	Deep learning	Black box	Integrated in HIS	keras

^1^Visits triaged Red/Orange/yellow are directed to regular emergency department patient Flow and Green/Blue visits are directed to fast track. HIS – hospital’s information system

^2^Tree depth, complexity, and the use of variables with many categories was taken into consideration to keep the tree simple and therefore applicable in the fast-paced context of triage

The ML models tested have very different characteristics and some have parameters that are set in advance and control various aspects of the training process itself i.e. hyperparameters. These hyperparameters can be changed, and the performance of the models evaluated in a process called hyperparameter tunning. As an example, and considering decision trees, cost complexity is used for pruning the tree to avoid overfitting. It adjusts the trade-off between accuracy (and possible overfitting) and tree simplicity.

The default threshold of 0.5 for binary classification was kept for all the models.

### Data preparation and model training

The dataset was randomly split 20% for testing and 80% for training with stratification for the outcome. On the training set, it was used a 2-fold cross validation with stratification for the outcome, repeated 20 times.

All categorical missing values were imputed the category “unknown”, there were no missing numerical data. Only the variables “Pretriage discriminator” and “Pretriage discriminator group” had an expected, and relevant missing count (96.7%). These variables are only filled in particular circumstances during the triage algorithm. All other categorical variables had negligible (> 0.5%) of missing count.

For tensor flow model and the XGboost model, dummy variables were created i.e., one binary numeric variable was created for each category. Furthermore, the variable “Triage maincomplaint discriminator” was removed from the simple tree model because it has too many categories (217) and would be unusable in the paper-based approach. To mitigate complexly, the tree depth maximum was also set to 5 in hyper parameter tuning phase. The variable “Triage maincomplaint discriminator” was also removed from the logistic regression model, as some levels had only a small number of observations and the information was already aggregated in the variable “Main complaint group”.

All the data analysis was performed using R version 4.2.2 (2022–10-31) [23]. The integrated development environment (IDE) used was RStudio Version 2022.12.0 + 353 [24]. The ecosystem of packages “tidymodels” was used. The specific packages used for training the ML models are underlined in Table 1.

### Models’ evaluation

The evaluation metrics used in our study to compare the performance of different machine learning methods are enumerated below. Accuracy (accuracy), Negative Predictive Value (npv), Positive Predictive Value (ppv), Sensitivity (sens) Specificity (spec), Kappa (kap), Area Under Curve (roc_auc) and F-measure (F_meas) that combines ppv and sensitivity, providing a single score that reflects both aspects of a model's performance. Furthermore, False Positives (FP) i.e., non-avoidable visits classified as avoidable, False Negatives (FN) i.e. avoidable visits classified as non-avoidable, True Positives i.e. visits classified correctly as avoidable and True Negatives (TN) i.e. visits classified correctly non-avoidable were also computed for each model.

## Results

The dataset utilized for training and testing the models comprised a total of 507,708 visits to the pediatric emergency department. Of these visits 17.4% were referrals, and 4.4% resulted in hospital admissions. Females accounted for 46.8% of the visits. The average age of patients was 7 years, with a standard deviation of 5.5 years. The mean length of stay was 102.4 min, with a standard deviation of 154.5 min. Regarding the outcome, 41.6% of the visits were considered avoidable. Concerning triage levels, 0.2% were triaged level 1, 5.6% level 2, 39.4% level 3, 50.4% level 4 and 4.5% level 5.

### Models’ performance

Considering the metrics that evaluate the models globally i.e. accuracy, f_meas, kap and roc_auc, all ML models outperformed the classification made by triage rules i.e. visits triaged levels 1 (red), 2 (orange) and 3 (yellow) were considered non-avoidable and visits triaged levels 4 (green) and 5 (blue) were considered avoidable. Except for the classification made by triage rules, the models had similar performance, namely accuracy, ranging from 70 to 72% and AUCs ranging from 74 to 80%. However, regarding measures that evaluate the specific performance i.e., NPV, PPV, sensitivity and specificity, there is greater variation Moreover, the triage rules model was also outperformed by the all ML models in terms of measures addressing specific performance, except for sensitivity.

### Predictions on test data

**Fig. 2:**
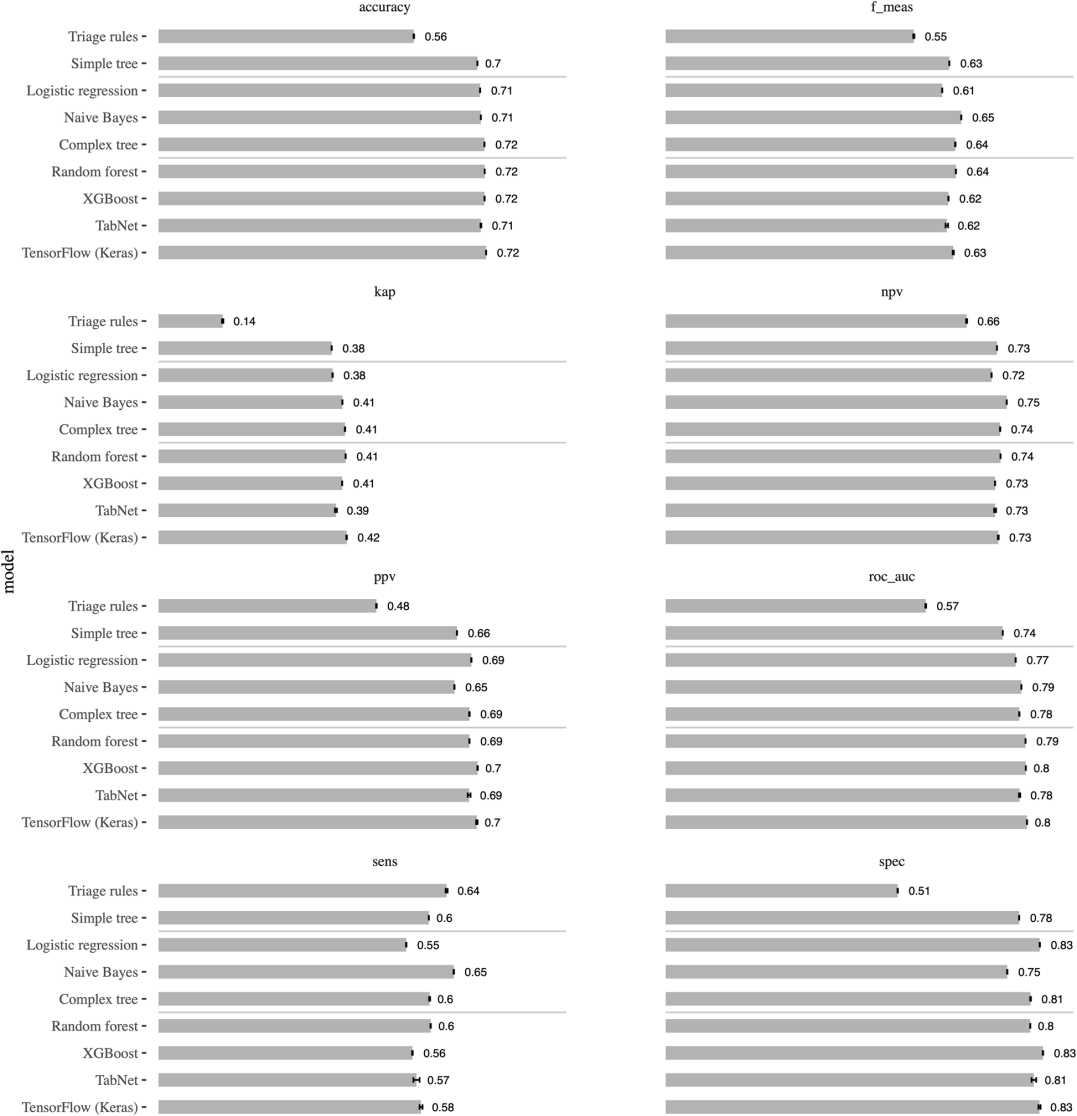
Bar plot with error bars, comparing the performance of the predictive models used to classify if visit to the Paediatrics emergency department is avoidable, and should be directed the fast-track area, by metric. Results were obtained from the twofold cross validation with stratification for the outcome, repeated 20 times

**Fig. 3:**
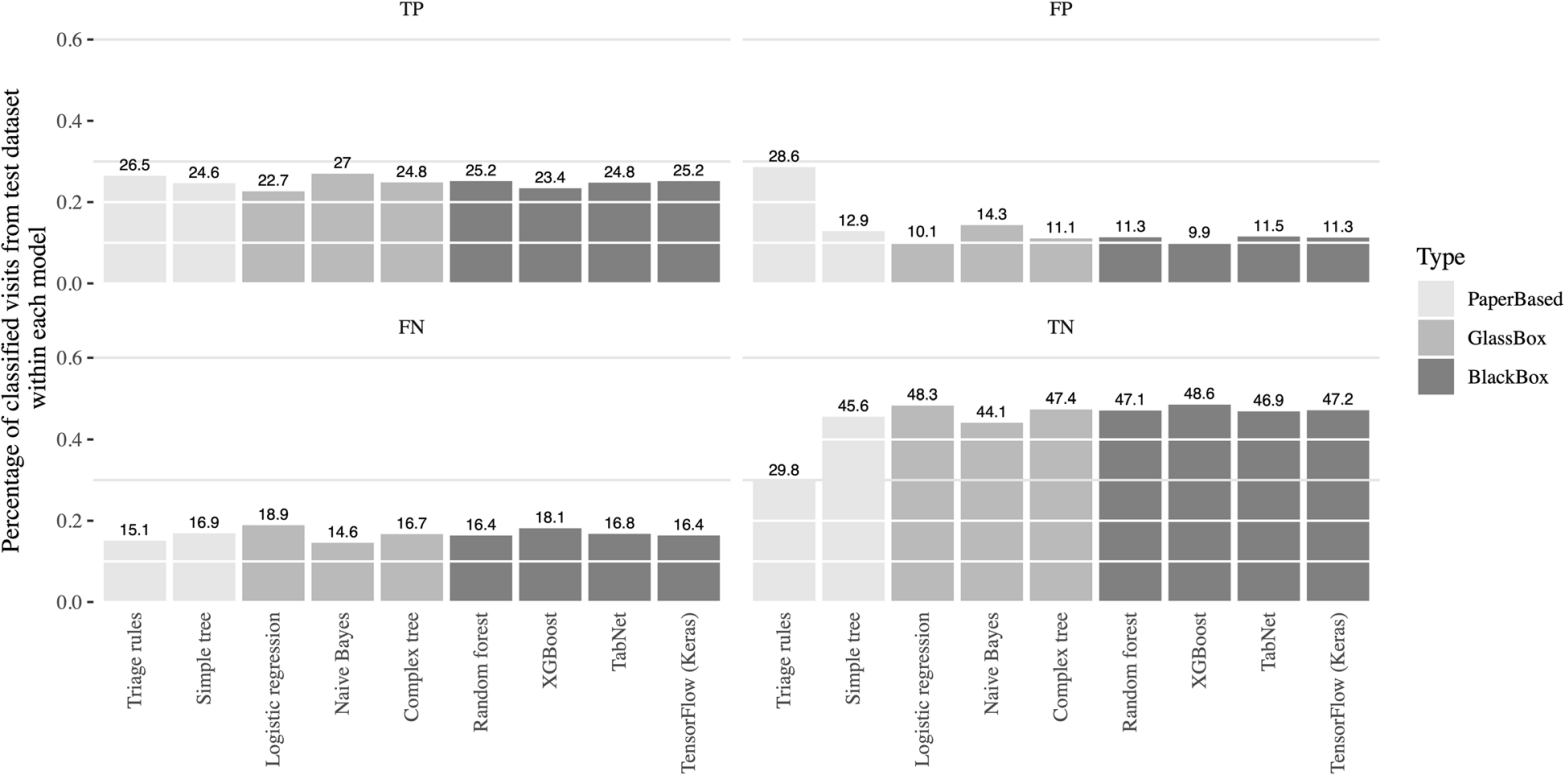
Percentage of visits, for each model, incorrectly assigned to the fast-track i.e., non-avoidable classified as avoidable (FP), incorrectly assigned to normal flow i.e. avoidable classified as non-avoidable (FN) and percentage of visits classified correctly, either avoidable (TP) or non-avoidable (TN). Data was obtained from the test dataset when the models were fitted

For a more pragmatic and in-depth analysis and drilling down from Figs. 2 and 3 presents a confusion matrix’s inspired plot, where the model’s classifications are compared. This visualization enables us to calculate, for a particular number of visits, how many are correctly classified and misclassified, for any given model. It is important to highlight the low FP proportion of all ML models and the high TN proportion, with relatively small differences between them. On the other hand, the classification made by triage rules had the highest FP proportion and the lowest TN proportion. The FN rate and the TP rate were relatively constant across all models.

### Simple classification tree

**Fig. 4:**
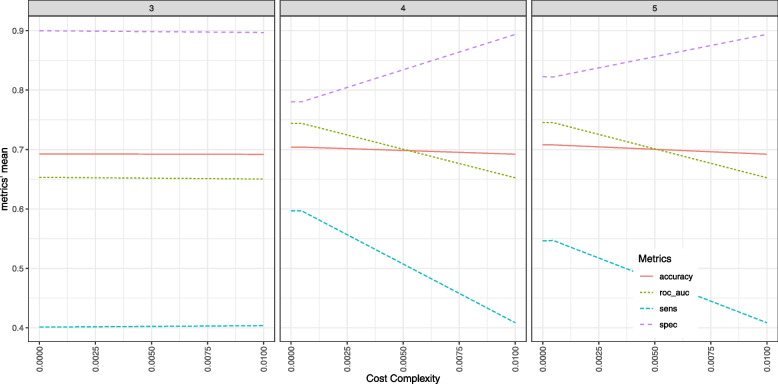
Plots with the results from a grid hyperparameter tunning for the simple decision tree. Each plot refers to a specific tree depth. Metric’s mean is the result of a twofold cross validation repeated 20 times

When constructing the model for the simple classification tree, the hyperparameter tunning was limited to a tree depth of 5. Fig. 4 shows that above a tree depth of 4 there is no significant improvement in overall performance i.e., accuracy and AUC. Furthermore, when the value of cost complexity decreases, sensitivity increases.

**Fig. 5:**
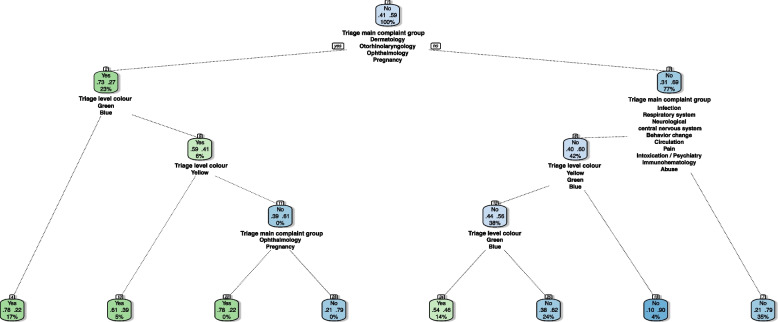
Decision tree with the hyperparameters set to tree depth of 4 and a cost complexity of 1e-4, for the classification of visits as avoidable (Yes) or non-avoidable (No), and redirection to a fast track or regular flow respectively, in the Paediatric emergency depart. This tree was created from the test dataset

Figure 5 presents a decision tree with the hyperparameters set to: tree depth of 4 and a cost complexity of 1e-4. This combination was chosen for the best balance between sensitivity and specificity, not having a significant impact on overall performance i.e., accuracy and AUC. This particular tree only used the triage’s “Main complaint group” and triage level.

## Discussion

The major objective of this study was to evaluate several ML models to be implemented in the PED and aid in the decision if a visit to the Paediatrics emergency department is avoidable and should be directed to the fast-track area or is non-avoidable and can stay in the regular flow.

To the best of our knowledge, the assignment of visits to a low acuity fast track is mostly done by the assigned triage levels and there are no studies with a pragmatic focus on implementation with data-driven approaches. [7–10, 16, 25, 26].

Our approach was to leverage the power of ML to aid in the assignment for the fast-track. First, creating an outcome based on resource utilization and PED discharge destination. And afterwards trying to predict it using information known at the time of triage.

### Summary of main findings

Regarding overall performance metrics, all ML models had similar performance and outperformed the classification made by triage rules. Drilling down, errors made by triage rules were mainly false positives, i.e. non avoidable patients sent to the fast track.

The pruned decision tree performed only slightly worse in the overall metrics than all the other ML models and their errors in classification went in the same direction as the other more complex models, as can be seen when analysing sensitivity, specificity or the confusion matrix.

### Results contextualization

This study's results regarding AUC were slightly higher than those found by Chang et al. who made a similar study using ML to identify low-severity patients. However, the definition of candidates for fast track was the time interval between the triage registry and being discharged within less than 4 h. Therefore, depending on the setting, this could be a problem, since the length of stay is greatly influenced by the triage level and the hour of the day. Hence, we think that this study's approach, only considering resource usage and discharge destination, appears less prone to confounders [27].

In a study by Kwon et al. [28] where the aim was to identify high-risk patients at the time of triage, the ML algorithm predicted in-hospital mortality, critical care, and hospitalization more accurately than existing triage systems. When considering only hospitalization, the results are very similar to our study [28]. Despite the different outcomes, these results support the superiority of ML models over triage system’s classification to predict outcomes in the ED. This is further reinforced and expanded by the results of a systematic review by Kareen et al. where it was found that in an ED setting, ML models outperformed usual care in most diagnostic and prognostic predictions across all studies [16].

It is essential to consider the FP values for each model, i.e., visits that the models classified as avoidable that really were not avoidable. While false negative (FN) proportion, i.e., visits that the models classified as non-avoidable that were avoidable, are directed to the previous patient flow, a high FP proportion might be a reason for concern since they are non-avoidable visits directed to the fast track. In this regard, the split made by triage rules is a reason for worry, since its FP proportion doubles the highest ML model. This highlights the necessity to include all relevant stakeholders (e.g. physicians, nurses, administrative personnel and patients) in the development of the DSS and is vital to its successful implementation, as only these stakeholders know the particularities of the context where the DSS is to be deployed. The ML models should be evaluated considering not only the performance metrics but also potential clinical consequences and impact on patient outcomes [16, 29].

### Implications for policy and practice

To discuss the choice of the model to be implemented is necessary to return to the three evaluation dimensions stated in the introduction: complexity, predictive performance, and interpretability. The pruned decision tree is very simple to implement. Based on two or three simple rules, a nurse at the end of triage could redirect the patient with minimal increased logistic burden. As a consequence of its simplicity, the time to implementation and cost is negligible. Regarding the performance assigning patients to a fast track based on triage levels is clearly not the best solution, given the high proportion of FP. Despite not being the best model, the pruned decision tree is outperformed by other more complex models but only by a few percentage points. The last dimension is extremely important and goes beyond interpretation, it relates the understanding to how the decision was made, and if the explanation is satisfactory, it builds trust [30]. And a trustworthy and explainable system has been a stakeholder requisite, especially in healthcare [31, 32]. In this regard, rules extracted from a low-depth decision tree excels.

The choice of the model was clear. The pruned decision tree could be implemented immediately, and the patients and staff could immediately reap the rewards of an improved PED workflow. The resources committed to the implementation are low, hence can be easily replaced if a better alternative is developed.

### Limitations

The definition of avoidable visit was chosen among others [6, 33, 34] for being triage system agnostic, better reflecting the visit’s lack of necessity for the hospital’s resources. Nevertheless, the multiple definitions used in the field make comparisons less accurate.

There are few clinical parameters available for model building. Nevertheless, if more variables were to be collected and used, the model's performance could never worsen.

The data gathered had the original purpose of providing care to the patients in the PED, therefore subjected to the bias of any observational study based on routinely collected data, e.g. information system downtime and the inability to control how the variables are collected.

The deployment phase will present challenges and model’s suggestions might have to be adapted the day-to-day operations and decision-making processes in clinical settings.

External validity of the models was not tested. However, given the intended use of the models, it's crucial to consider both the target population and the setting. Thus, if this study were to be conducted in different settings or with different populations, the models would need to be retrained with the data available [35].

## Conclusions

This study demonstrates the substantial potential of ML models to enhance decision-making processes in Emergency Departments, regarding the assignment of patients to appropriate care paths. Our findings underscore the superior performance of ML models over more traditional methods in determining the patient flow. While all tested ML models performed well, the pruned decision tree model emerged as a practical choice due to its simplicity, ease of implementation, and relatively high accuracy. Moreover, this model supports the need for interpretable and trustworthy systems in healthcare, as it allows healthcare providers to understand and trust the basis of its predictions. Moving forward, the integration of ML into clinical settings should continue to focus on balancing complexity, predictive performance, and interpretability, ensuring that such tools are not only technically effective but also align with the practical realities and ethical considerations of medical practice. Finally, the obtained results can potentially help to design patients’ flow management strategies in PED settings, which has been sought as a solution for addressing the long-standing problem of overcrowding.

More data-driven approaches, where the patient and healthcare professionals are put first and technology serves an instrumental role in solving the problem, are necessary in this age of AI hype. Small, targeted interventions to solve real-world problems with real-world data are paramount to the future of healthcare.

## Data Availability

The data that support the findings of this study are available from the hospital, but restrictions apply to the availability of these data, which were used under hospital’s authorization for the current study, and so are not publicly available. Data are however available from the authors upon reasonable request and authorized by the hospital’s administration.

## Abbreviations

ED: Emergency Department
DSS: Decision Support Systems
ML: Machine Learning
EHR: Electronic Health Records
PED: Paediatric Emergency Department
HIS: Hospital's Information System
CSV: Comma Separated Values
NPV: Negative Predictive Value
PPV: Positive Predictive Value
Sens: Sensitivity
Spec: Specificity
Kap: Kappa
ROC_AUC: Area Under Curve
PaedCTAS: Canadian Triage and Acuity Scale paediatric guidelines
F_meas: F-measure
TNR: True Negative Rate
TPR: True Positive Rate
TP: True Positive
TN: True Negative
FP: False Positive

## References

[1] H. R. Rasouli et al., “Outcomes of Crowding in Emergency Departments; a Systematic Review,” Arch. Acad. Emerg. Med., vol. 7, no. 1, Aug. 2019. Available: https://www.ncbi.nlm.nih.gov/pmc/articles/PMC6785211/. Accessed: Apr. 30, 2021. PMC6785211 31602435

[2] Chan M, Meckler G, Doan Q. Paediatric emergency department overcrowding and adverse patient outcomes. Paediatr Child Health. 2017;22(7):377–81. 10.1093/pch/pxx111. 29479252 10.1093/pch/pxx111 PMC5804927

[3] Doan Q, et al. The impact of pediatric emergency department crowding on patient and health care system outcomes: a multicentre cohort study. CMAJ Can Med Assoc J J Assoc Med Can. 2019;191(23):E627–35. 10.1503/cmaj.181426. 10.1503/cmaj.181426 PMC6565395 31182457

[4] Wiler JL, et al. Optimizing emergency department front-end operations. Ann Emerg Med. 2010;55(2):142–160.e1. 10.1016/j.annemergmed.2009.05.021. 19556030 10.1016/j.annemergmed.2009.05.021

[5] Oredsson S, et al. A systematic review of triage-related interventions to improve patient flow in emergency departments. Scand J Trauma Resusc Emerg Med. 2011;19:43. 10.1186/1757-7241-19-43. 21771339 10.1186/1757-7241-19-43 PMC3152510

[6] Parkinson B, Meacock R, Checkland K, Sutton M. Clarifying the concept of avoidable emergency department attendance. J Health Serv Res Policy. 2021;26(1):68–73. 10.1177/1355819620921894. 32517553 10.1177/1355819620921894 PMC7734604

[7] Arya R, Wei G, McCoy JV, Crane J, Ohman-Strickland P, Eisenstein RM. Reasing length of stay in the emergency department with a split emergency severity index 3 patient flow model. Acad Emerg Med. 2013;20(11):1171–9. 10.1111/acem.12249. 24238321 10.1111/acem.12249

[8] Kwa P, Blake DF. Fast track: has it changed patient care in the emergency department? Emerg Med Australas. 2008;20(1):10–5. 10.1111/j.1742-6723.2007.01021.x. 17999686 10.1111/j.1742-6723.2007.01021.x

[9] Sanchez M, Smally AJ, Grant RJ, Jacobs LM. Effects of a fast-track area on emergency department performance. J Emerg Med. 2006;31(1):117–20. 10.1016/j.jemermed.2005.08.019. 16798173 10.1016/j.jemermed.2005.08.019

[10] O’Brien D, Williams A, Blondell K, Jelinek GA. Impact of streaming ‘fast track’ emergency department patients. Aust Health Rev. 2006;30(4):525–32. 10.1071/ah060525. 17073548 10.1071/ah060525

[11] Martin HA, Noble M, Wilmarth J. Improving patient flow and decreasing patient length of stay in the Pediatric Emergency Department through implementation of a fast track. Adv Emerg Nurs J. 2021;43(2):162–9. 10.1097/TME.0000000000000351. 33915567 10.1097/TME.0000000000000351

[12] Vance J, Sprivulis P. Triage nurses validly and reliably estimate emergency department patient complexity. Emerg Med Australas. 2005;17(4):382–6. 10.1111/j.1742-6723.2005.00761.x. 16091102 10.1111/j.1742-6723.2005.00761.x

[13] Feretzakis G, et al. Using machine learning techniques to predict hospital admission at the emergency department. J Crit Care Med. 2022;8(2):107–16. 10.2478/jccm-2022-0003. 10.2478/jccm-2022-0003 PMC9097643 35950158

[14] Miles J, Turner J, Jacques R, Williams J, Mason S. Using machine-learning risk prediction models to triage the acuity of undifferentiated patients entering the emergency care system: a systematic review. Diagn Progn Res. 2020;4:16. 10.1186/s41512-020-00084-1. 33024830 10.1186/s41512-020-00084-1 PMC7531169

[15] Mueller B, Kinoshita T, Peebles A, Graber MA, Lee S. Artificial intelligence and machine learning in emergency medicine: a narrative review. Acute Med Surg. 2022;9(1):e740. 10.1002/ams2.740. 35251669 10.1002/ams2.740 PMC8887797

[16] Kareemi H, Vaillancourt C, Rosenberg H, Fournier K, Yadav K. Machine learning versus usual care for diagnostic and prognostic prediction in the emergency department: a systematic review. Acad Emerg Med. 2021;28(2):184–96. 10.1111/acem.14190. 33277724 10.1111/acem.14190

[17] Raita Y, Goto T, Faridi MK, Brown DFM, Camargo CA, Hasegawa K. Emergency department triage prediction of clinical outcomes using machine learning models. Crit Care. 2019;23:64. 10.1186/s13054-019-2351-7. 30795786 10.1186/s13054-019-2351-7 PMC6387562

[18] “Statistics Portugal. Instituto Nacional de Estatística - Portugal.” Accessed: Feb. 26, 2020. Available: https://www.ine.pt

[19] Warren DW, et al. Revisions to the Canadian Triage and Acuity Scale paediatric guidelines (PaedCTAS). CJEM. 2008;10(3):224–43. 19019273 10.1017/S1481803500010149

[20] Benchimol EI, et al. The Reporting of studies conducted using observational routinely-collected health data (RECORD) statement. PLOS Med. O. 2015;12(10):e1001885. 10.1371/journal.pmed.1001885. 26440803 10.1371/journal.pmed.1001885 PMC4595218

[21] Chen T, Guestrin C. “XGBoost: a scalable tree boosting system,” in proceedings of the 22nd ACM SIGKDD international conference on knowledge discovery and data mining, in KDD ’16. New York, USA: Association for Computing Machinery; 2016. p. 785–94. 10.1145/2939672.2939785.

[22] Arik SÖ, Pfister T. “TabNet: attentive interpretable tabular learning,” Proc. AAAI Conf. Artif. Intell. 2021;35(8). 10.1609/aaai.v35i8.16826.

[23] “R: The R Project for Statistical Computing.” Accessed: Aug. 31, 2021. Available: https://www.r-project.org/

[24] “RStudio | Open source & professional software for data science teams.” Accessed: Aug. 31, 2021. Available: https://rstudio.com/

[25] Sánchez-Salmerón R, et al. Machine learning methods applied to triage in emergency services: a systematic review. Int Emerg Nurs. 2022;60:101109. 10.1016/j.ienj.2021.101109. 34952482 10.1016/j.ienj.2021.101109

[26] Fernandes M, Vieira SM, Leite F, Palos C, Finkelstein S, Sousa JMC. Clinical decision support systems for triage in the emergency department using intelligent systems: a review. Artif Intell Med. J. 2020;102:101762. 10.1016/j.artmed.2019.101762. 31980099 10.1016/j.artmed.2019.101762

[27] Chang Y-H, et al. Machine learning–based triage to identify low-severity patients with a short discharge length of stay in emergency department. BMC Emerg Med. 2022;22(1):88. 10.1186/s12873-022-00632-6. 35596154 10.1186/s12873-022-00632-6 PMC9123815

[28] Kwon J, Lee Y, Lee Y, Lee S, Park H, Park J. Validation of deep-learning-based triage and acuity score using a large national dataset. PLoS ONE. 2018;13(10):e0205836. 10.1371/journal.pone.0205836. 30321231 10.1371/journal.pone.0205836 PMC6188844

[29] F. S. van Royen, F. W. Asselbergs, F. Alfonso, P. Vardas, and M. van Smeden, “Five critical quality criteria for artificial intelligence-based prediction models,” Eur. Heart J., p. ehad727, Oct. 2023, 10.1093/eurheartj/ehad727. 10.1093/eurheartj/ehad727 PMC10702458 37897346

[30] P. Madumal, T. Miller, L. Sonenberg, and F. Vetere, “Explainable Reinforcement Learning Through a Causal Lens.” arXiv, Nov. 20, 2019. 10.48550/arXiv.1905.10958.

[31] Combi C, et al. A manifesto on explainability for artificial intelligence in medicine. Artif Intell Med. N. 2022;133:102423. 10.1016/j.artmed.2022.102423. 36328669 10.1016/j.artmed.2022.102423

[32] Langer M, et al. What do we want from Explainable Artificial Intelligence (XAI)? – A stakeholder perspective on XAI and a conceptual model guiding interdisciplinary XAI research. Artif Intell. J. 2021;296:103473. 10.1016/j.artint.2021.103473. 10.1016/j.artint.2021.103473

[33] Mistry RD, Brousseau DC, Alessandrini EA. Urgency classification methods for emergency department visits: do they measure up? Pediatr Emerg Care. 2008;24(12):870–4. 10.1097/PEC.0b013e31818fa79d. 19092571 10.1097/PEC.0b013e31818fa79d

[34] Hsia RY, Niedzwiecki M. Avoidable emergency department visits: a starting point. Int J Qual Health Care. 2017;29(5):642–5. 10.1093/intqhc/mzx081. 28992158 10.1093/intqhc/mzx081

[35] Sperrin M, Riley RD, Collins GS, Martin GP. Targeted validation: validating clinical prediction models in their intended population and setting. Diagn Progn Res. 2022;6(1):24. 10.1186/s41512-022-00136-8. 36550534 10.1186/s41512-022-00136-8 PMC9773429

